# Sublingual Immunization with a Live Attenuated Influenza A Virus Lacking the Nonstructural Protein 1 Induces Broad Protective Immunity in Mice

**DOI:** 10.1371/journal.pone.0039921

**Published:** 2012-06-27

**Authors:** Hae-Jung Park, Boris Ferko, Young-Ho Byun, Joo-Hye Song, Gye-Yeong Han, Elisabeth Roethl, Andrej Egorov, Thomas Muster, Baiklin Seong, Mi-Na Kweon, Manki Song, Cecil Czerkinsky, Huan H. Nguyen

**Affiliations:** 1 International Vaccine Institute, Seoul, Korea; 2 AVIR Green Hills Biotechnology AG, Vienna, Austria; 3 Department of Biotechnology and Translational Research Center for Protein Function Control, Yonsei University, Seoul, Korea; The Ohio State University, United States of America

## Abstract

The nonstructural protein 1 (NS1) of influenza A virus (IAV) enables the virus to disarm the host cell type 1 IFN defense system. Mutation or deletion of the NS1 gene leads to attenuation of the virus and enhances host antiviral response making such live-attenuated influenza viruses attractive vaccine candidates. Sublingual (SL) immunization with live influenza virus has been found to be safe and effective for inducing protective immune responses in mucosal and systemic compartments. Here we demonstrate that SL immunization with NS1 deleted IAV (DeltaNS1 H1N1 or DeltaNS1 H5N1) induced protection against challenge with homologous as well as heterosubtypic influenza viruses. Protection was comparable with that induced by intranasal (IN) immunization and was associated with high levels of virus-specific antibodies (Abs). SL immunization with DeltaNS1 virus induced broad Ab responses in mucosal and systemic compartments and stimulated immune cells in mucosa-associated and systemic lymphoid organs. Thus, SL immunization with DeltaNS1 offers a novel potential vaccination strategy for the control of influenza outbreaks including pandemics.

## Introduction

Infection with influenza type A viruses causes annual epidemics with potential to develop into pandemics affecting hundreds millions worldwide. Vaccination against influenza is a key tool for controlling influenza epidemics and pandemics. Currently, only intramuscular (IM) formaldehyde and propionolactone-inactivated and IN cold-adapted attenuated vaccines are licensed in humans [see reviews [1], [2], [3]]. The efficacy of both types of vaccines has been reported to be comparable in adults [4]. However, live-attenuated influenza vaccines (LAIV), apart from the convenience of their administration appear to induce longer-lasting and broader cross-protective immunity than corresponding inactivated vaccines [4], [5], [6], [7], [8], [9].

Although cold-adapted influenza vaccines (CAIVs) are safe and approved for human use the precise genetic and molecular mechanisms of attenuation based on single mutations are not completely understood [10], [11] CAIVs are capable to replicate in humans and especially in children for several days [12]. Genetic stability of CAIV is hard to predict since viruses re-isolated from immunized hosts often reveal a different set of point mutations as compared to that of original vaccine viruses [12]. An alternative approach based on reverse genetics to obtain influenza viruses containing modifications in the NS1 gene has been developed [13]. NS1 deleted viruses (DeltaNS1) lacking the NS1 protein-dependent alpha/beta interferon (IFN-α/β) antagonist function [13], [14] are genetically stable and are replication-deficient in IFN-competent hosts. Importantly, such viruses are capable of inducing protection in mice [15], [16], ferrets and non-human primates [17]. Moreover, DeltaNS1 vaccine candidate is well-tolerated, safe and immunogenic in healthy volunteers [18]. Due to the lack of the entire NS1 cistron in DeltaNS1 viruses, this mutation cannot be compensated for by any suppressor mutation and, unlike LAIV, DeltaNS1 virus re-isolation from immunized hosts is rare and at most limited to early time-points after immunization supporting the notion that replication of DeltaNS1 virus is essentially abortive [18].

Delivery of LAIV via the IN (spray, drops) and pulmonary (aerosol delivery) routes targets the nasopharynx-associated lymphoid tissue (NALT) and the lung mucosa, respectively. Such formulations induce protective immunity against influenza virus [7], [19]. However, post-licensure surveillance studies of a nasal killed influenza vaccine adjuvanted with *Escherichia coli* heat-labile enterotoxin identified a possible association with rare but severe cases of Bell's palsy [20]. The sublingual (SL) (under the tongue) route has recently received attention as an attractive site for delivery of drugs because proteins and/or peptides are not subjected to the degradation as opposed to oral administration that delivers agents directly to the upper gastrointestinal tract. SL delivery of antigen has proven effective for administering protein allergens [21]. Recently, we have shown that administration of inactivated and even live influenza virus via the SL route did not redirect the viruses to the central nervous system (CNS) [22]. Thus, the SL route confers substantial safety advantages for mucosal delivery of influenza virus vaccines. Furthermore, SL administration of non-replicating antigens, including inactivated influenza virus induces broad-spectrum immune responses in the airway and genital mucosa, as well as in extra-mucosal tissues (blood, peripheral lymph nodes, and spleen) [22], [23], [24]. The induced immune responses comprise serum and secretory antibody (Ab) responses and pulmonary effector cytotoxic T lymphocyte (CTL) responses. Importantly, SL is effective in inducing so called heterosubtypic immunity (HSI), the cross-protection against infection by a subtype different from the immunizing one [22]. Although initial antigen uptake by SL dendritic cells (DC) and their subsequent migration and antigen-presentation occur in draining cervical lymph nodes (CLN) [25], the inductive mechanisms of SL immunization remain to be elucidated.

It is generally accepted that live-attenuated vaccines induce better HSI than injectable killed vaccines [4], [5], [6], [7], [8], [9], [26], [27]. Attempts to generate vaccines that include IAV conserved proteins for induction of HSI have been made [28], [29], [30], [31], but no HSI vaccine is currently available. In this study we evaluated the effect of a novel vaccination strategy that combines a newly developed replication-deficient influenza virus - DeltaNS1 - and an alternative mucosal delivery route - SL - for induction of broad protection against infection with different influenza virus subtypes. The mechanisms by which SL immunization with influenza induces specific immune responses in different lymphoid organs were explored.

## Materials and Methods

### DeltaNS1 Viruses

Generation of A/PR8 DeltaNS1 (DeltaH1N1) virus was described in detail elsewhere [14], [16]. To obtain a high virus titer infected Vero cell supernatant was ultrracentrifuged (Beckmann L80 Ultracentifuge SW 32Ti rotor) at 32,000 rpm (125,750×g) through a 30% sucrose/SPGN buffer cushion (30% sucrose; 75 mM NaCl; 3,8 mM KH_2_PO_4;_ 7,2 mM K_2_HPO_4_, 4,9 mM L-Glutamat; pH 7,5) for 2 hr at 4°C and the purified virus pellet was resuspended in SPGN buffer containing 6% sucrose. The final virus titer was 10^9,4^ TCID_50/_ml.

The A/Vietnam/1203/04 DeltaNS1 (Delta H5N1) virus was obtained by reverse genetics in Vero cells [17], as a 5∶3 reassortant, encoding four proteins, namely hemagglutinin (HA), neuraminidase (NA), M1, and M2 of the A/Vietnam/1203/04 (H5N1) virus while the remaining genes were derived from the IVR-116 reassortant vaccine strain distributed by the World Health Organization (WHO).The IVR-116 inherited HA and NA genes from influenza A/New Caledonia/20/99 (H1N1), the PB1 gene from A/Texas/1/77 (H3N2), and all other genes from the A/Puerto Rico/8/34 (H1N1) (PR8) virus. The HA cleavage site was modified in a trypsin-dependent manner complementing the deleted NS1 gene as an attenuation factor. The DelNS1 H5N1 virus was able to grow in serum-free Vero cell cultures to a high titer of 10^8,2^ TCID_50/_ml. Both delNS1 viruses were replication-deficient in interferon-competent cells and were not shed in immunized animals.

### Viruses

Egg-grown influenza virus strains A/PR/8/34 (H1N1) (A/PR8), A/Philippines/2/82/X-79 (H3N2) (A/Philippines) were prepared as previously reported [32]. Mouse-adapted viruses A/PR8 and A/Philippines prepared as lung homogenates of IN infected mice were used for challenge as previously described [32]. The A/Aquatic bird/Korea/W81/2005 (H5N2), isolated from wild bird in Korea in 2006, kindly provided by Dr. Young-Ki Choi, Chungbuk University, Korea, was adapted by multiple passages (15 times) in BALB/c mice. After final passage, single plaque was isolated by three consecutive plaque purifications on MDCK cells, amplified in embryonated chicken eggs, and the LD_50_ of the H5N2 virus was determined in mice for challenge experiment.

### Animals

Female BALB/c mice aged 6–8 weeks were purchased from Orient Bio Inc. (Orient Inc, Korea). All mice were maintained in specific pathogen–free barrier facilities. A/PR8-hemagglutinin (HA-TCR) transgenic mice, prepared as previously described [33], were bred in International Vaccine Institute animal facility. All experiments and animal procedures conformed to protocols approved by the Institutional Animal Care and Use Committees at International Vaccine Institute (2010-017) and Yonsei University (2010-00-32619), Seoul, Korea.

### Cell Lines

Madin-Darby canine kidney (MDCK) cells (ATCC, Manassas, VA), were maintained in standard complete Dulbecco's modified Eagle's medium (D-MEM) (Gibco, Grand Island, NY) containing 5% fetal bovine serum (FBS) and antibiotics.

### Immunization

For SL immunization, 5 µl of vaccine were placed underneath the tongue of ketamine-anesthetized mice using micropipette. The procedure was repeated 4 times in 5-minute interval to deliver the total of 20 µl of vaccine per mouse per immunization. Mice were kept with heads placed in ante flexion for 30 minutes after the immunization before being returned back to the cages. For IN and nasal (N) immunization, each ketamine-anesthetized mouse received a total of 50 µl (25 µl per each nostril) or 10 µl (5 µl per each nostril) of vaccine, respectively. The vaccine doses are specified in Figure legends.

### Infection with Influenza Viruses

Fifty percent lethal dose (LD_50_) was determined by inoculating groups of eight mice intranasally with serial 10-fold dilutions of virus as previously described [34]. For infection, ketamine-anesthetized mice were inoculated intranasally with a lethal dose with 5×LD_50_ of A/PR/8/34 (H1N1), A/Philippines (H3N2), or A/Aquatic bird/Korea/W81/2005 (H5N2) viruses resuspended in 50 µl PBS per animal.

### Hemagglutination-inhibition (HI) Assay

Samples were treated with receptor destroying enzyme II (RDE, Denka Seiken Co., Ltd., Tokyo, Japan) at a final dilution of 1∶3 before being tested in HI assay. Two-fold serially diluted samples were incubated with equal volume containing 100 TCID_50_ of viruses in V shaped-bottom 96-well microtiter plates at 37°C for 1 h. At the end of incubation, freshly prepared 1% chicken red blood cells (CRBC) were added, and plates were mixed by agitation, covered, and allowed to set for 1 h at 4°C temperature. The HI titers were determined by the reciprocal of the last dilution which contained non-agglutinated CRBC. Positive and negative control samples were included on each plate.

### ELISA

The standard ELISA was performed for detection of virus-specific Abs in the sera and secretions. 96-well MaxiSorp^TM^ Nunc-Immuno plates (Nalgene Nunc International, Naperville, IL) were coated overnight with inactivated virus at a concentration of 5 µg/ml. Dilutions of specimens were incubated 2 h on coated and blocked ELISA plates. Bound immunoglobulins were detected with goat anti-mouse Ig (H+L) horseradish peroxidase-conjugated antibody (Southern Biotechnologies Associates, Inc., Birmingham, AL). At the end of the incubation (2 h at 37°C), tetramethylbenzidine (TMB) substrate was added and the reaction was stopped with an equal volume of 1 M sulfuric acid. The color developed was measured in a SPECTRAmax photometer at 450 nm. The reproducibility of the assay was ascertained by applying a control hyperimmune mouse serum on each plate. Assay results were expressed as end-point titration values which are determined by the last dilutions that are above cutoff for assay (OD 450 nm reaches plateau).

### In vivo Lymphocyte Proliferation Assay

Hemagglutinin (HA)-specific CD4^+^ T cells were isolated from all lymph nodes of HA-TCR transgenic mice of BALB/c background using MACS beads (Miltenyi Biotec, Auburn, CA). The HA-specific CD4^+^ T cells were labeled with CFSE (Invitrogen, Carlsbad, CA) according to manufacturer’s manual. A total of 1.8×10^7^ CFSE labeled cells were intravenously transferred to BALB/c. Next day the recipients were immunized sublingually with 2×10^7^ pfu of Delta H1N1, 2×10^5^ pfu of mouse-adapted wt live or 40 µg of formalin-inactivated A/PR8. Three days later, proliferation of transferred CD4^+^ T cells isolated from different lymphoid tissues was visualized by FACS analysis of their CFSE profile on BD FACS Calibur Flow Cytometer (BD Biosciences, San Jose, CA).

### Statistics

The data are expressed as the mean ± one standard error of the mean (SEM) and compared using a two-tailed student’s *t-*test or an unpaired Mann Whitney U test available in Microsoft Excel software (Redmond, WA).

## Results

### SL Immunization with DeltaNS1 Viruses Induces Protection against Homotypic and Heterosubtypic Challenge

Two live-attenuated influenza A virus vaccine candidates that have deleted nonstructural protein NS1 gene, the A/Vietnam/1203/04 DeltaNS1 (Delta H5N1) and A/PR8 DeltaNS1 (Delta H1N1) mutants, were examined in this study.

**Figure 1:**
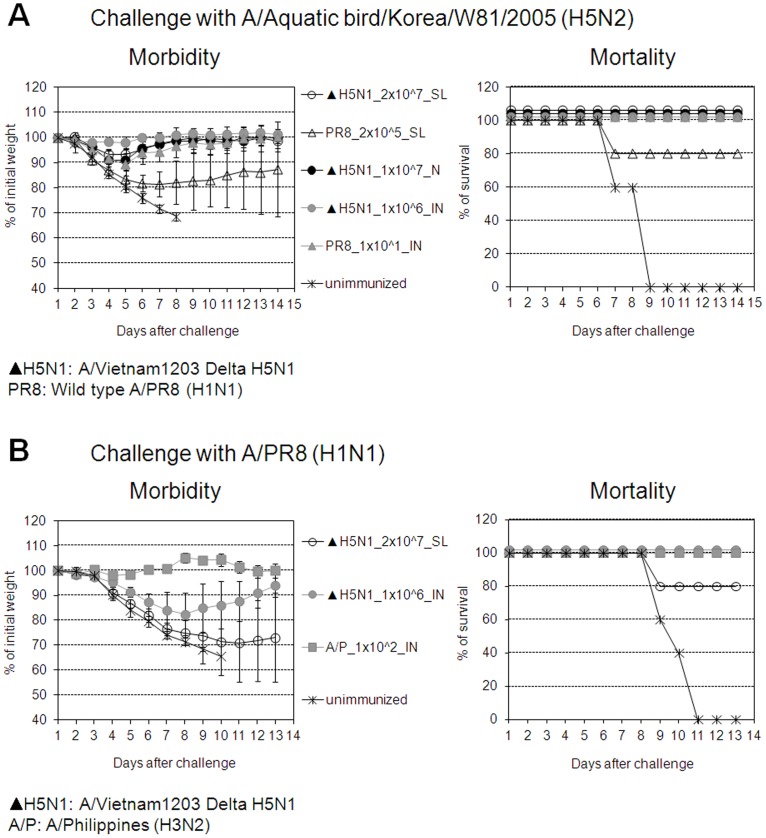
SL Immunization with Delta H5N1 induces homotypic and heterosubtypic immunity. BALB/c mice were immunized with different doses of Delta H5N1 (▴ H5N1) or wt live virus A/PR8/34 (PR8) H1N1 via the sublingual (SL), nasal (N) or intranasal (IN) route. Four weeks later, the mice were IN challenged with homotypic A/Aquatic bird/Korea/W81/2005 (H5N2) (Fig. 1A) or heterosubtypic A/PR8 (H1N1) (Fig. 1B) as described in Materials and Methods. Morbidity and mortality were monitored daily for at least 3 weeks. Mean body weight ± SD of each experimental group of 5–10 mice was determined at each indicated time point. Mortality is expressed as mean % ± SD of mice that survived the challenge. The values are the means of 5–10 mice per group.

**Figure 2:**
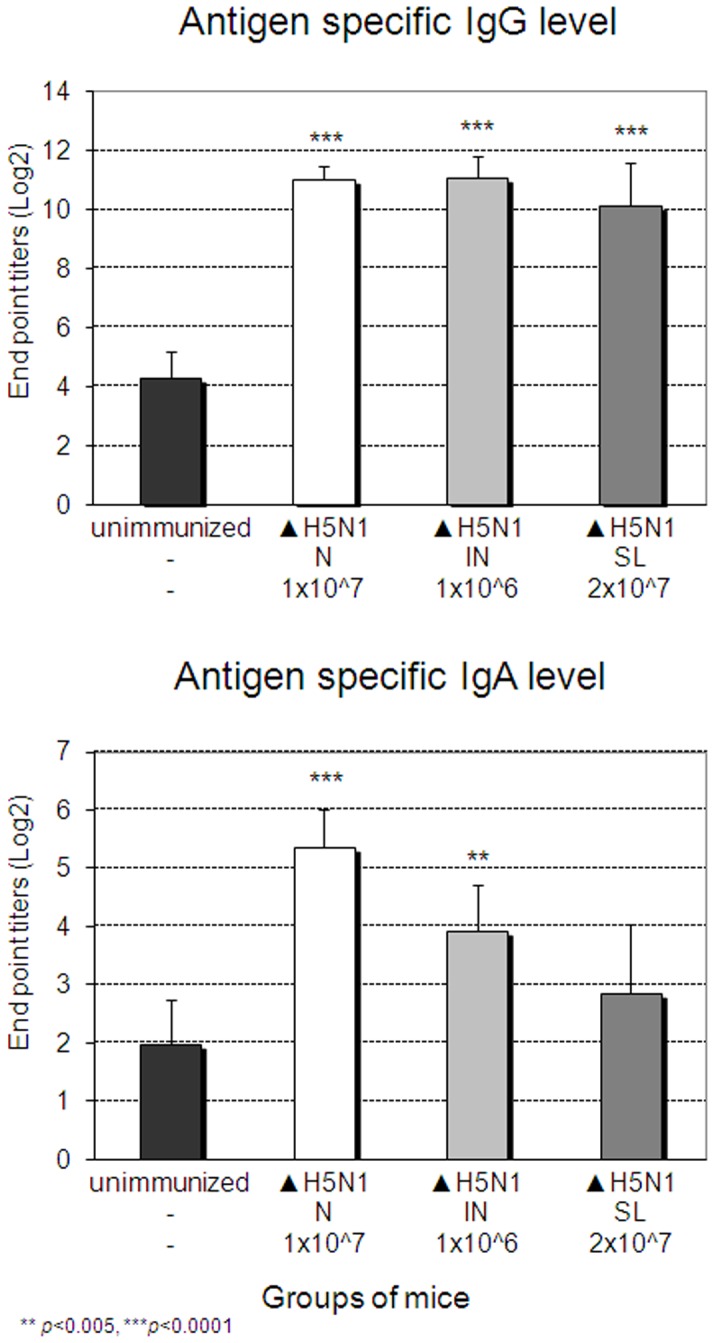
Induction of virus-specific IgG and IgA upon immunization with Delta H5N1. BALB/c mice were immunized with different doses of Delta H5N1 (▴ H5N1) or wt live virus A/PR8/34 (PR8) H1N1 via the sublingual (SL), nasal (N) or intranasal (IN) route. Four weeks later sera were collected and the levels of H5N1 virus-specific IgG and IgA were determined by Delta H5N1 virus-coated ELISA plates. The values represent the mean + SEM (vertical bars) end point ELISA antibody titers determined on 5 mice per group.

We first tested the immunogenicity of Delta H5N1 vaccine candidate as it would be stockpiled for use in the event of H5N1 influenza outbreak. As shown in Figure 1, a single SL immunization with Delta H5N1 subtype induced complete protection against infection with mouse-adapted A/Aquatic bird/Korea/W81/2005 (H5N2) which shares 94.4% nucleotide sequence homology with HA of highly pathogenic avian influenza virus A/VN1203 (H5N1) and Delta H5N1 viruses (Fig. 1A). The level of protection is comparable with that seen in mice immunized with the vaccine given through IN route. When lethally challenged with heterosubtype A/PR8 H1N1, 80% of Delta H5N1 immunized survived (Fig.1B). Of note, NAs of A/PR8 H1N1 and A/VN1203 H5N1 share 84% amino acid sequence identity and considered significantly drifted. Results suggest that single SL immunization with replication-deficient influenza A viruses H5N1 lacking the NS1 gene induced complete protection against challenge with drifted virus and high level of HSI. Protection was associated with antigen-specific IgG Ab levels in blood (Fig. 2). Although the dose used for SL immunization was significantly higher than that used for N or IN immunization, it is important that SL immunization with DelNS1 viruses induced antigen specific IgG at levels comparable to those induced by administration of the vaccine to different sites of the respiratory tract, a finding which is consistent with earlier reports [32], [35].

**Figure 3:**
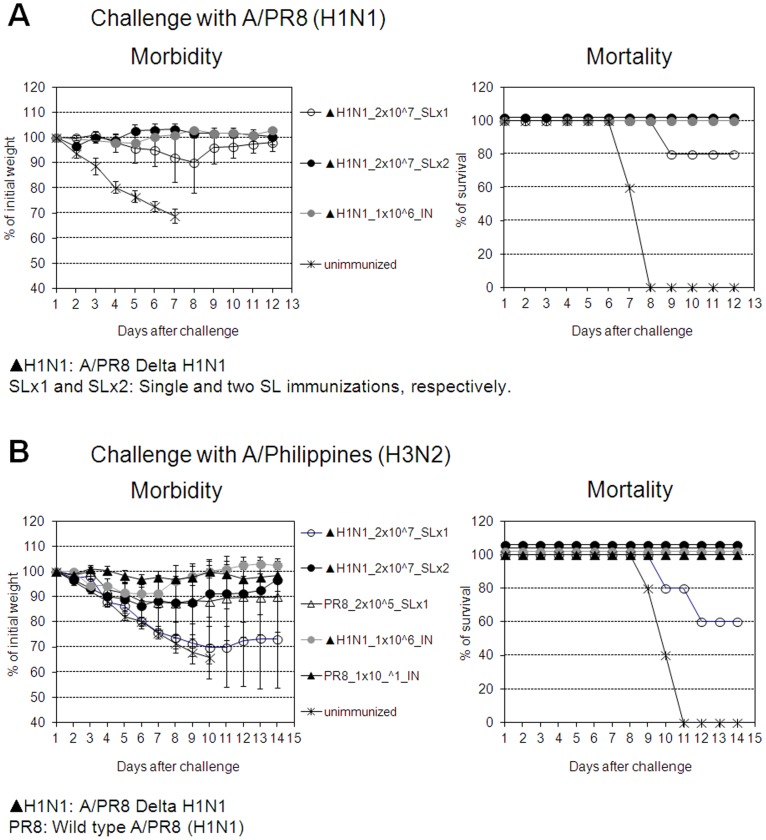
Induction of homotypic and HSI upon immunization with Delta H1N1. BALB/c mice were immunized IN or SL with different doses of Delta H1N1 (▴H1N1), given once (1x) or twice (2x) at 2-week intervals. Four weeks later, animals were intranasally challenged with 5×LD_50_ of homologous mouse-adapted A/PR8 (Fig. 3A) or heterosubtypic mouse-adapted A/Philippines (A/P) H3N2 (Fig. 3B) virus. Morbidity and mortality were monitored daily. Data are expressed as mean (body weight or % survival) determined on groups of 5–10 mice.

**Figure 4:**
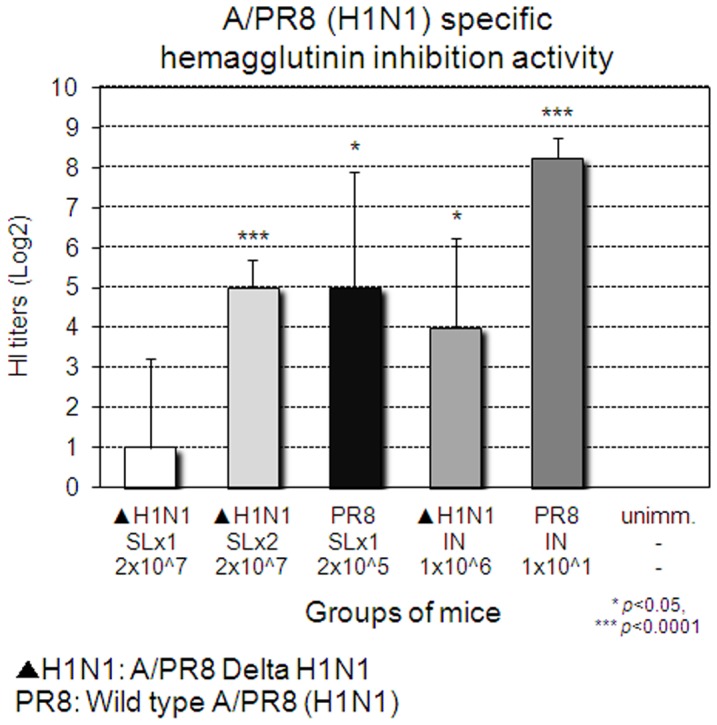
Induction of HI Abs upon immunization with Delta H1N1. On the day before challenge as described in Figure 3, the sera were collected and the titers of HI Abs were determined against A/PR8 virus. The values represent the mean + SEM antibody titers of sera from 5 mice per group.

**Figure 5:**
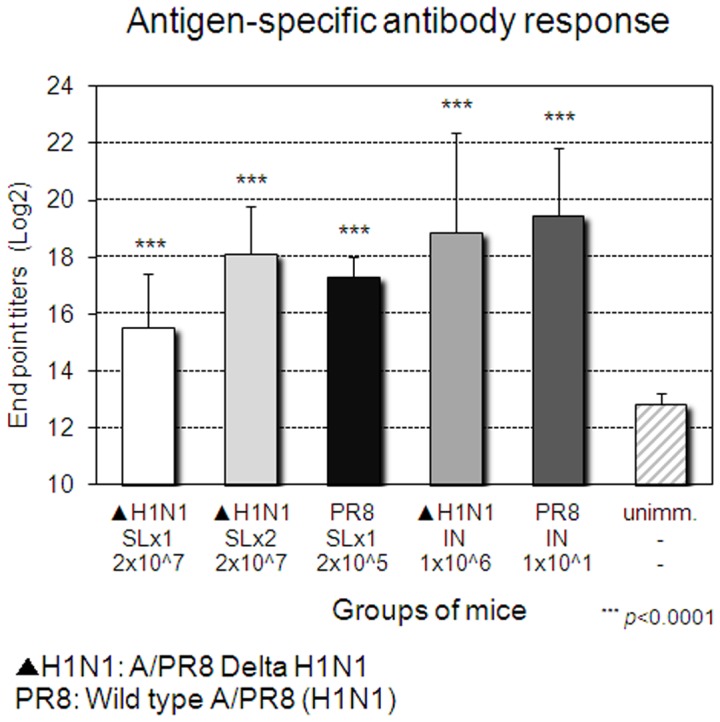
Induction of virus-specific Abs upon immunization with Delta H1N1. On the day before challenge as described in Figure 3, the sera were collected and the titers of virus-specific Abs were determined by A/PR8 virus-coated ELISA plates. The values represent the mean + SEM antibody titers of sera from 5 mice per group.

Next, we examined the breadth of protection induced by A/PR8 Delta H1N1 vaccine since A/PR8 (H1N1) and A/Philippines (H3N2) viruses are common laboratory mouse-adapted influenza strains. Four weeks after a single SL immunization with Delta H1N1, mice were challenged with homologous A/PR8 (H1N1) or heterosubtypic A/Philippines (H3N2) virus. As shown in Fig. 3A, 80% of SL immunized mice survived a lethal challenge with mouse-adapted homologous A/PR8 (H1N1). When challenged with heterosubtypic A/Philippine (H3N2) viruses, 60% of immunized mice recovered from the lethal infection (Fig. 3B). However, complete protection (100%) against homotypic and heterosubtypic challenges was readily achieved by a SL booster immunization given two weeks after primary immunization (Fig. 3A and 3B). Likewise, SL or IN immunization with a sublethal dose of mouse-adapted virus A/PR8 (H1N1), or IN immunization with Delta H1N1 induced complete protection against homotypic and heterosubtypic challenge, in keeping with previous reports [17], [22], [32]. Thus, either a single IN immunization or 2 SL immunizations with Delta H1N1 induced complete protective homotypic and HSI. Protection was associated with levels of HI Abs in blood (Fig. 4) and virus-specific Abs (Fig. 5), as the second SL immunization with Delta H1N1 boosted effectively Ab responses to levels comparable to those induced by wild type (wt) viruses or IN immunization with Delta H1N1.

### SL Immunization with DeltaNS1 IAV Induces Mucosal and Systemic Ab Responses

**Figure 6:**
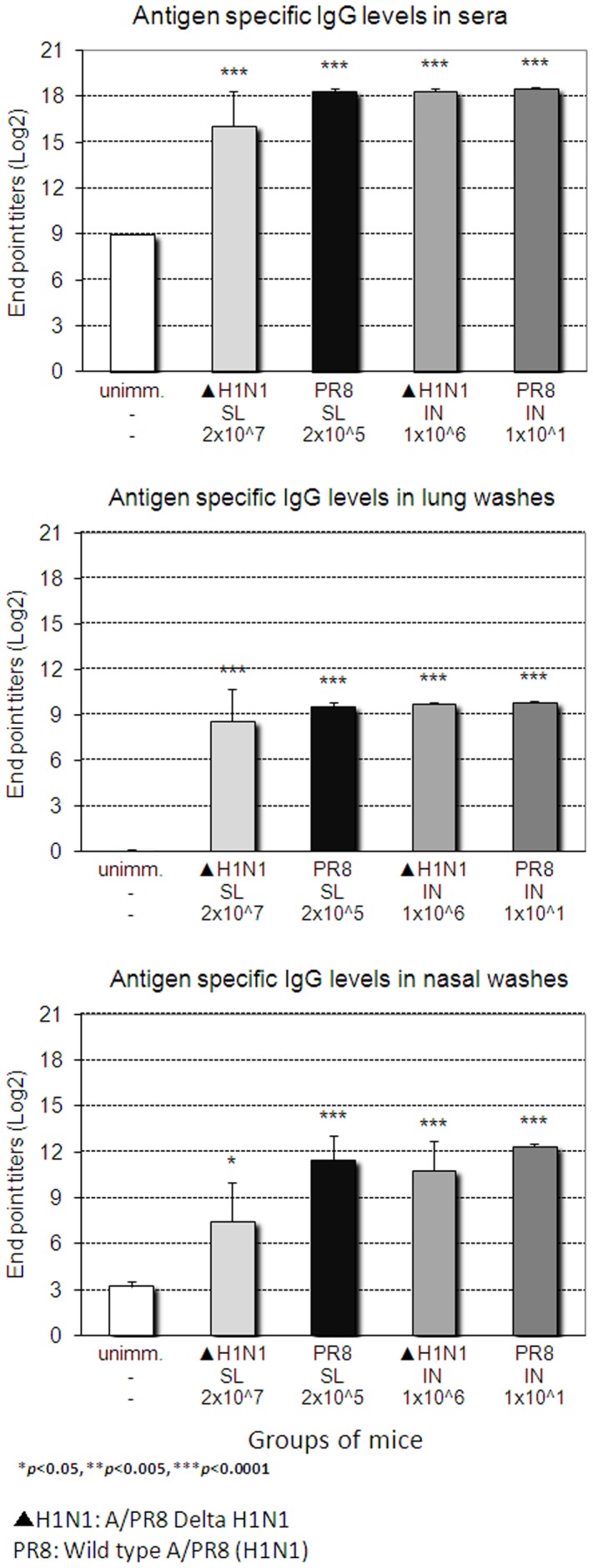
Levels of virus-specific IgG induced in mucosal and systemic compartments upon immunization with DeltaNS1 IAV. BALB/c mice were immunized with different doses of Delta H1N1 (▴ H1N1) or wt live virus A/PR8/34 (PR8) H1N1 (WT) via the sublingual (SL) or intranasal (IN) route. Four weeks later, titers of H1N1 (A/PR8) virus-specific IgG in sera and secretions were determined by ELISA. The values represent mean + SEM ELISA titers determined on groups of 5 mice.

**Figure 7:**
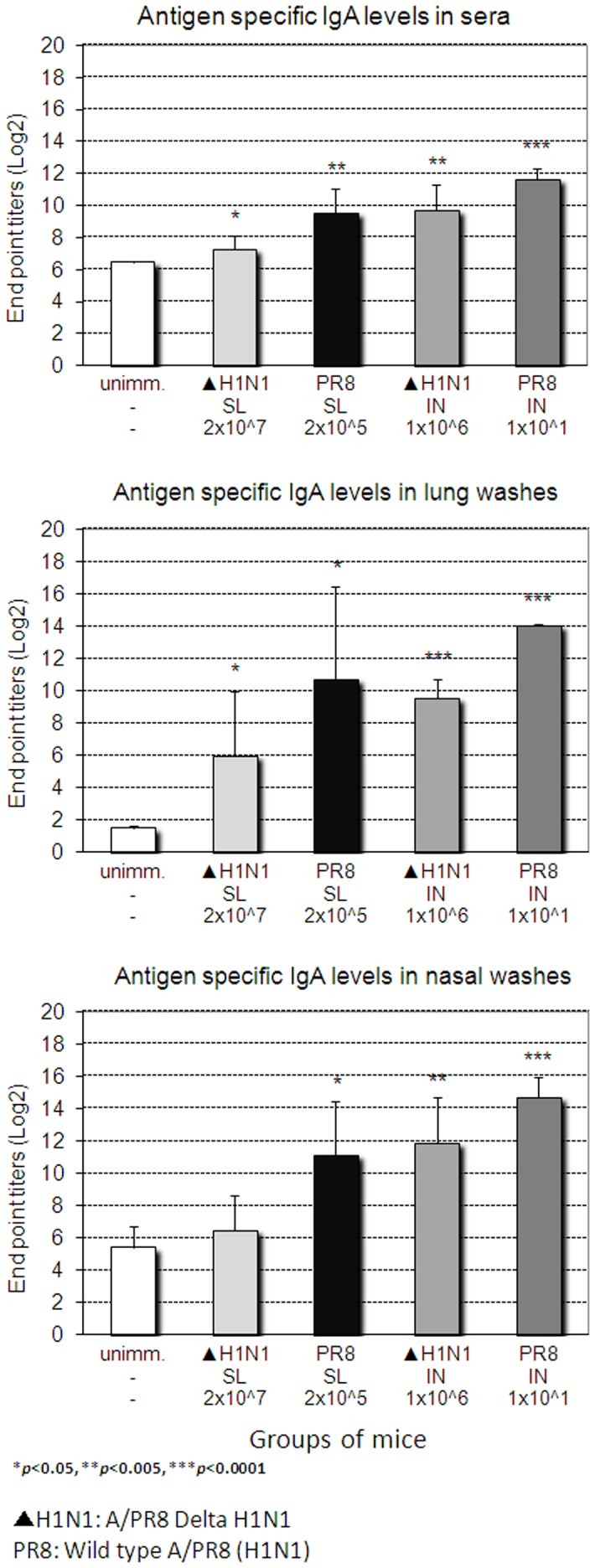
Levels of virus-specific IgA induced in mucosal and systemic compartments upon immunization with DeltaNS1 IAV. BALB/c mice were immunized as described in Figure 6. Four weeks later, titers of H1N1 (A/PR8) virus-specific IgA in sera and secretions were determined by ELISA. The values represent mean + SEM ELISA titers determined on groups of 5 mice.

Since Ab responses play an important role in protection against challenge with same subtype by virus neutralization as well as in HSI [32], [36], we examined whether SL immunization with live attenuated DeltaNS1 IAV induced virus-specific antibody responses. As shown in Fig. 6, single SL immunization with live attenuated DeltaNS1 IAV (H1N1) induced significant levels of virus-specific IgG in blood, lungs and nasal washes 4 weeks after immunization. SL immunization induced lower levels of virus-specific IgG in blood and nasal washes as compared to IN immunization with the same vaccine or SL immunization with a sublethal dose of wt live virus. However, levels of virus-specific IgG induced in the lungs after SL immunization were comparable to those induced by other immunization routes with the same vaccine or immunization with a sublethal dose of wt live virus. Virus-specific IgA levels induced by SL immunization with DeltaNS1 IAV were lower compared to those induced by N or IN immunization with the same vaccine or SL immunization with a sublethal dose of wt live virus (Fig. 7). Of note, levels of virus-specific IgA induced in the lungs but neither in the nasal cavity nor in blood correlated with protection. These results indicate that the virus-induced specific Ab levels provide a correlate of vaccine efficacy against lethal challenge.

### SL Immunization with DeltaNS1 IAV Activates Immune Cells in Mucosa-associated and Systemic Lymphoid Organs

**Figure 8:**
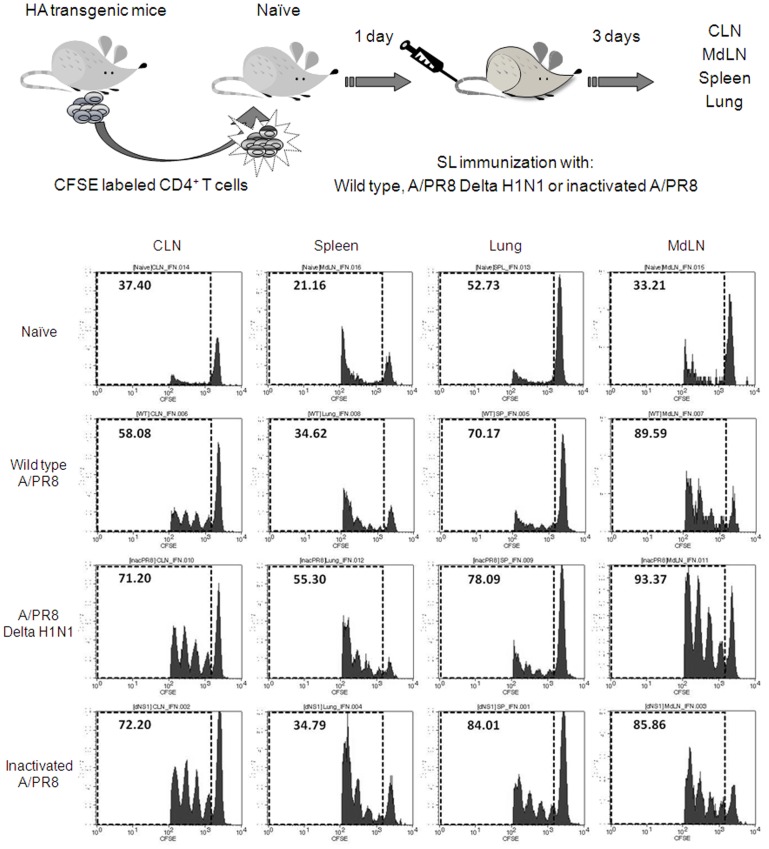
SL immunization with DelNS1 vaccine activates mucosal and extramucosal CD4+ T cells. CFSE-labeled CD4^+^ T cells from HA-TCR transgenic mice were adoptively transferred into naïve mice. One day later, recipients were given SL 2×10^7^ pfu of Delta H1N1, 2×10^5^ pfu of mouse-adapted wt live or 40 µg of formalin-inactivated A/PR8. Three days after the immunization, proliferating (CFSE stained) HA-TCR CD4^+^ T cells were detected in cervical lymph nodes (CLN), lungs, mediastinal lymph nodes (MdLN) and spleens by FACS analysis. The data are representative of two experiments showing similar results.

To better understand the mechanisms whereby SL immunization induces broad mucosal and systemic immune responses, we conducted adoptive transfer experiments as schematically illustrated in Figure 8. CD4^+^ T cells isolated from HA-TCR transgenic mice were labeled with CFSE and adoptively transferred to naïve mice. One day later, recipients were given a single SL dose of Delta H1N1. Three days after the immunization, the proliferative activity of mononuclear cells from lungs, cervical lymph nodes (CLN), mediastinal (MdLN) and spleens was analyzed by flow cytometry. Proliferating HA-TCR transgenic CD4^+^ T cells were observed in all lymph nodes analyzed, including MdLN. The proliferative activitiy of lymph node cells was comparable in SL immunized mice given either wt live virus or inactivated vaccine. These results demonstrate that SL DeltaNS1 IAV efficiently stimulates immune cells in local and distant mucosa-associated and systemic lymphoid organs resulting in initiation and recall of immune responses in these tissues.

## Discussion

In this study we provide proof-of-concept that a novel influenza vaccination strategy using the SL route for delivery of live-attenuated replication-deficient influenza virus that lacks the entire NS1 gene induced protective immunity against infection by the same or a different virus subtype. Indeed, we found that a single SL immunization readily induced protection against infection with the same subtype, however two immunizations were required for inducing complete protection against challenge by a different subtype. Protection was comparable to that seen after infection with a sublethal dose of wt virus. These results are consistent with our previous findings showing that SL immunization with a sublethal dose of wt live virus induced both homotypic and HSI [22]. In the current study, partial HSI induced by a single SL immunization suggests that virus replication competence may play a role in protection induced by the vaccine. It is interesting that Delta H5N1 seems to be more effective for inducing protective immunity against heterosubtypic H1N1 virus challenge. The finding is supported by recent study showing that hen egg yolk Abs (IgY) raised against H5N1 whole inactivated virus recognize conserved epitopes present in both virus types H5N1 and H1N1 and provide cross-protection against infection with H1N1 virus. In contrast, IgY raised against H1N1 whole inactivated virus recognize only type-specific protective epitopes that are capable of protecting only against different strains of H1N1 but not against H5N1 [37]. The molecular basis of these findings is not clear and should be addressed in future studies.

Protection induced by SL Delta NS1 virus was associated with serum HI Ab levels and virus-specific Ab titers in systemic and mucosal compartments. Even within a group of mice that received a single SL immunization, only animals who developed high levels of serum virus-specific Abs survived a lethal challenge (data not shown). Moreover, a single SL immunization with DeltaNS1 virus induced incomplete and variable levels of protection against homologous and heterologous challenges. These observations indicate that SL application of liquid vaccine may not consistently distribute virus particles onto the SL mucosa and/or could be influenced by the presence variable amounts of saliva from animal to animal. Although SL immunization required significantly higher doses of the vaccine given at relatively higher concentrations (application volumes are limited to a few µl) to achieve levels of protective immunity comparable to IN immunization, our findings indicate that SL immunization is an attractive option for non-invasive delivery of DeltaNS1 vaccine. Further studies in primates and humans are warranted, especially given the fact that live attenuated vaccines may show comparable immunogenicity after oral or IN administration in humans [38]. Optimal formulation of the vaccine for SL application needs to be developed to facilitate consistent retention and uptake of the virus while reducing the dose.

In addition, SL DeltaNS1 vaccine was as immunogenic and protective as mouse-adapted live virus given by the same route. Furthermore, SL immunization with DeltaNS1 induced broad virus-specific IgG and IgA Ab responses in blood, lung and nasal washes. The latter findings are in keeping with our previous observations that the SL mucosa is an efficient site for induction of broad-spectrum mucosal and systemic immune responses [23], [24] to a variety of antigens, including live or inactivated influenza virus [22].

In an attempt to elucidate the mechanisms governing induction of protective immune responses by SL immunization, we conducted a study on the role of resident antigen-presenting cells (APC) as potential factors in initiating immunity to SL antigens. We have shown previously that briefly after SL administration, dendritic cells (DC) in SL mucosa captured the antigen and migrated to CLN, where they share the processed antigen with resident DC to initiate antigen-specific immune responses. The process is dependent on CCR7-CCL19/CCL21 pathway [25]. In current study we adapted adoptive transfer experiments and found that SL administration of DeltaNS1 virus stimulates antigen-specific proliferation of immune cells in local and distant mucosa-associated and systemic lymphoid tissues. This was also true for both inactivated and wt live viruses. These findings suggest that the SL mucosa is an effective mucosal immune inductive site and that resident APCs in SL mucosa play an important role in the initiation and recall of broad immune responses. The precise role for SL DC subsets in induction of broad immune response is being investigated in our laboratory.

It is unlikely that SL administration of our live attenuated replication-deficient DeltaNS1 vaccine redirects the vaccine virus to the CNS since neither inactivated nor live virus was detected in olfactory bulbs after SL immunization [22]. The safety of SL immunization is further supported by our observation that SL application of as many as 2×10^5^ pfu of mouse-adapted A/PR8 virus (4,000 LD_50_) did not induce appreciable body weight loss or mortality in mice. In marked contrast, all mice succumbed to an IN application of as few as 250 pfu of the virus (5LD_50_). The safety of the CAIV is based on a large number of point mutations distributed across the internal gene segments. However, only a small number of mutations localized in the polymerase genes are responsible for the attenuation of cold-adapted virus strains that are unable to replicate at normal body temperature [10], [11]. The genetic stability of CAIV strains or other promising temperature-sensitive vaccine candidate strains is often questioned since viruses re-isolated from vaccinated hosts reveal additional point mutations that might eventually function as "suppressor" mutations even causing enhanced replication properties and a possible loss of the temperature-sensitive phenotype [11], [13], [39], [40], [41]. On the other hand, DeltaNS1 vaccine construct is genetically stable and replication-deficient in IFN-competent hosts [16], [18]. Obviously, a large deletion or lack of the entire NS1 cistron (delNS1virus) cannot be compensated for by any suppressor mutation. The high immunogenicity of live replication-deficient vaccines combined with the lack of vaccine virus shedding makes the DeltaNS1 virus a promising influenza vaccine candidate.

Thus, our study demonstrates that SL administration of DeltaNS1 vaccine, aside from being safe and easy to administer, can induce broad heterosubtypic immunity against influenza A viruses of different subtypes. This novel vaccination strategy based on SL administration of highly immunogenic replication-deficient DeltaNS1 virus does not require special delivery devices nor trained healthcare personnel and hence offers a promising option for the control of influenza outbreaks including pandemics.

## References

[1] Palese P (2006). Making better influenza virus vaccines?. Emerg Infect Dis.

[2] Palese P (2004). Influenza: old and new threats.. Nat Med.

[3] Palese P, Garcia-Sastre A (2002). Influenza vaccines: present and future.. J Clin Invest.

[4] Beyer WE, Palache AM, de Jong JC, Osterhaus AD (2002). Cold-adapted live influenza vaccine versus inactivated vaccine: systemic vaccine reactions, local and systemic antibody response, and vaccine efficacy. A meta-analysis.. Vaccine.

[5] Belshe RB, Gruber WC, Mendelman PM, Cho I, Reisinger K (2000). Efficacy of vaccination with live attenuated, cold-adapted, trivalent, intranasal influenza virus vaccine against a variant (A/Sydney) not contained in the vaccine.. J Pediatr.

[6] Belshe RB, Gruber WC, Mendelman PM, Mehta HB, Mahmood K (2000). Correlates of immune protection induced by live, attenuated, cold-adapted, trivalent, intranasal influenza virus vaccine.. J Infect Dis.

[7] Belshe RB, Mendelman PM, Treanor J, King J, Gruber WC (1998). The Efficacy of Live Attenuated, Cold-Adapted, Trivalent, Intranasal Influenzavirus Vaccine in Children.. N Engl J Med.

[8] Cox RJ, Brokstad KA, Ogra P (2004). Influenza virus: immunity and vaccination strategies. Comparison of the immune response to inactivated and live, attenuated influenza vaccines.. Scand J Immunol.

[9] Gorse GJ, Otto EE, Powers DC, Chambers GW, Eickhoff CS (1996). Induction of mucosal antibodies by live attenuated and inactivated influenza virus vaccines in the chronically ill elderly.. J Infect Dis.

[10] Herlocher ML, Clavo AC, Maassab HF (1996). Sequence comparisons of A/AA/6/60 influenza viruses: mutations which may contribute to attenuation.. Virus Res.

[11] Herlocher ML, Maassab HF, Webster RG (1993). Molecular and biological changes in the cold-adapted "master strain" A/AA/6/60 (H2N2) influenza virus.. Proc Natl Acad Sci U S A.

[12] Buonagurio DA, O'Neill RE, Shutyak L, D'Arco GA, Bechert TM (2006). Genetic and phenotypic stability of cold-adapted influenza viruses in a trivalent vaccine administered to children in a day care setting.. Virology.

[13] Egorov A, Brandt S, Sereinig S, Romanova J, Ferko B (1998). Transfectant influenza A viruses with long deletions in the NS1 protein grow efficiently in Vero cells.. J Virol.

[14] Garcia-Sastre A, Egorov A, Matassov D, Brandt S, Levy DE (1998). Influenza A virus lacking the NS1 gene replicates in interferon-deficient systems.. Virology.

[15] Talon J, Salvatore M, O'Neill RE, Nakaya Y, Zheng H (2000). Influenza A and B viruses expressing altered NS1 proteins: A vaccine approach.. Proc Natl Acad Sci U S A.

[16] Ferko B, Stasakova J, Romanova J, Kittel C, Sereinig S (2004). Immunogenicity and protection efficacy of replication-deficient influenza A viruses with altered NS1 genes.. J Virol.

[17] Romanova J, Krenn BM, Wolschek M, Ferko B, Romanovskaja-Romanko E (2009). Preclinical evaluation of a replication-deficient intranasal DeltaNS1 H5N1 influenza vaccine.. PLoS One.

[18] Wacheck V, Egorov A, Groiss F, Pfeiffer A, Fuereder T (2010). A novel type of influenza vaccine: safety and immunogenicity of replication-deficient influenza virus created by deletion of the interferon antagonist NS1.. J Infect Dis.

[19] Zuercher AW, Coffin SE, Thurnheer MC, Fundova P, Cebra JJ (2002). Nasal-associated lymphoid tissue is a mucosal inductive site for virus-specific humoral and cellular immune responses.. J Immunol.

[20] Mutsch M, Zhou W, Rhodes P, Bopp M, Chen RT (2004). Use of the inactivated intranasal influenza vaccine and the risk of Bell's palsy in Switzerland.. N Engl J Med.

[21] Agostinis F, Tellarini L, Canonica GW, Falagiani P, Passalacqua G (2005). Safety of sublingual immunotherapy with a monomeric allergoid in very young children.. Allergy.

[22] Song JH, Nguyen HH, Cuburu N, Horimoto T, Ko SY (2008). Sublingual vaccination with influenza virus protects mice against lethal viral infection.. Proc Natl Acad Sci U S A.

[23] Cuburu N, Kweon MN, Song JH, Hervouet C, Luci C (2007). Sublingual immunization induces broad-based systemic and mucosal immune responses in mice.. Vaccine.

[24] Cuburu N, Kweon MN, Hervouet C, Cha HR, Pang YY (2009). Sublingual immunization with nonreplicating antigens induces antibody-forming cells and cytotoxic T cells in the female genital tract mucosa and protects against genital papillomavirus infection.. J Immunol.

[25] Song JH, Kim JI, Kwon HJ, Shim DH, Parajuli N (2009). CCR7-CCL19/CCL21-regulated dendritic cells are responsible for effectiveness of sublingual vaccination.. J Immunol.

[26] Meitin CA, Bender BS, Small PA (1991). Influenza immunization: intranasal live vaccinia recombinant contrasted with parenteral inactivated vaccine.. Vaccine.

[27] Liang S, Mozdzanowska K, Palladino G, Gerhard W (1994). Heterosubtypic immunity to influenza type A virus in mice. Effector mechanisms and their longevity.. J Immunol.

[28] Neirynck S, Deroo T, Saelens X, Vanlandschoot P, Jou WM (1999). A universal influenza A vaccine based on the extracellular domain of the M2 protein..

[29] Mozdzanowska K, Feng J, Eid M, Kragol G, Cudic M (2003). Induction of influenza type A virus-specific resistance by immunization of mice with a synthetic multiple antigenic peptide vaccine that contains ectodomains of matrix protein 2.. Vaccine.

[30] Epstein SL, Kong WP, Misplon JA, Lo CY, Tumpey TM (2005). Protection against multiple influenza A subtypes by vaccination with highly conserved nucleoprotein.. Vaccine.

[31] Tompkins SM, Zhao ZS, Lo CY, Misplon JA, Liu T (2007). Matrix protein 2 vaccination and protection against influenza viruses, including subtype H5N1.. Emerg Infect Dis.

[32] Nguyen HH, van Ginkel FW, Vu HL, McGhee JR, Mestecky J (2001). Heterosubtypic immunity to influenza A virus infection requires B cells but not CD8+ cytotoxic T lymphocytes.. J Infect Dis.

[33] Kirberg J, Baron A, Jakob S, Rolink A, Karjalainen K (1994). Thymic selection of CD8+ single positive cells with a class II major histocompatibility complex-restricted receptor.. The Journal of experimental medicine.

[34] Maines TR, Lu XH, Erb SM, Edwards L, Guarner J (2005). Avian influenza (H5N1) viruses isolated from humans in Asia in 2004 exhibit increased virulence in mammals.. Journal of virology.

[35] Song SK, Moldoveanu Z, Nguyen HH, Kim EH, Choi KY (2007). Intranasal immunization with influenza virus and Korean mistletoe lectin C (KML-C) induces heterosubtypic immunity in mice.. Vaccine.

[36] Tumpey TM, Renshaw M, Clements JD, Katz JM (2001). Mucosal delivery of inactivated influenza vaccine induces B-cell-dependent heterosubtypic cross-protection against lethal influenza A H5N1 virus infection.. J Virol.

[37] Wallach MG, Webby RJ, Islam F, Walkden-Brown S, Emmoth E (2011). Cross-protection of chicken immunoglobulin Y antibodies against H5N1 and H1N1 viruses passively administered in mice.. Clinical and vaccine immunology : CVI.

[38] Aleksandrova GI, Smorodintsev AA, Beljaeva NM, Vasil'ev BJ, Geft RA (1970). Testing the safety and effectiveness of oral administration of a live influenza vaccine.. Bulletin of the World Health Organization.

[39] Romanov Iu R, Egorov A, Lisovskaia KV, Medvedeva TE, Nevedomskaia GN (1989). [The effect of amplifying reproduction of influenza virus in mouse lungs during simultaneous infection with two cold-adapted strains].. Vopr Virusol.

[40] Treanor J, Perkins M, Battaglia R, Murphy BR (1994). Evaluation of the genetic stability of the temperature-sensitive PB2 gene mutation of the influenza A/Ann Arbor/6/60 cold-adapted vaccine virus.. J Virol.

[41] Treanor JJ, Buja R, Murphy BR (1991). Intragenic suppression of a deletion mutation of the nonstructural gene of an influenza A virus.. J Virol.

